# Supplementary material for: Identification of Novel Coloboma Candidate Genes through Conserved Gene Expression Analyses across Four Vertebrate Species
Source: Biomolecules. 2023 Feb 4;13(2):293. doi: 10.3390/biom13020293 (PMC9953556; doi:10.3390/biom13020293)
Supplement: Supplementary file 1 [file biomolecules-13-00293-s001.zip › biomolecules-2146911-supplementaryfigures.pdf]

a

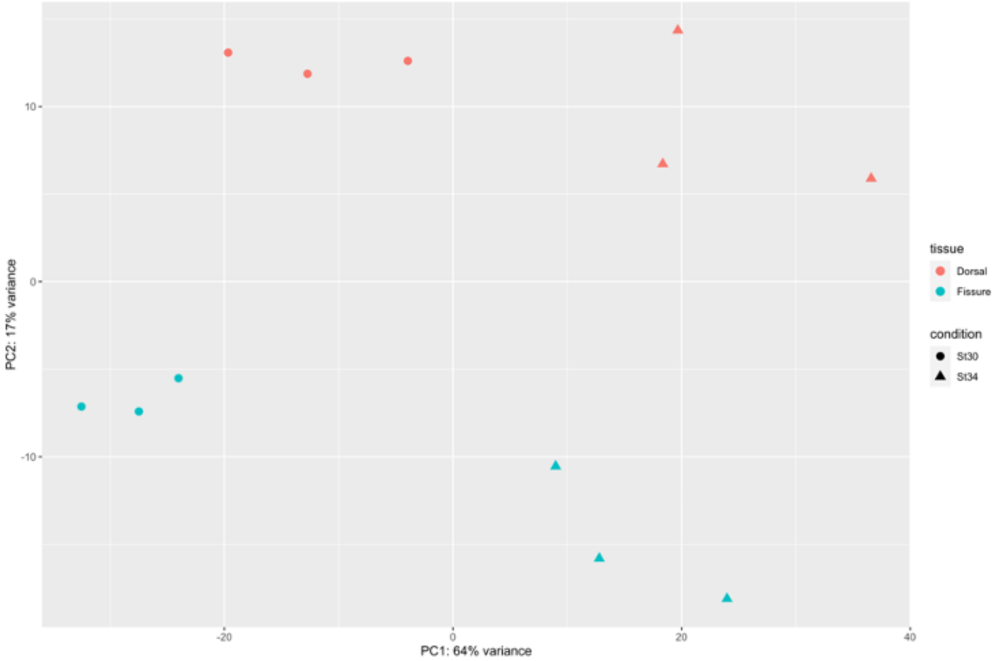

b

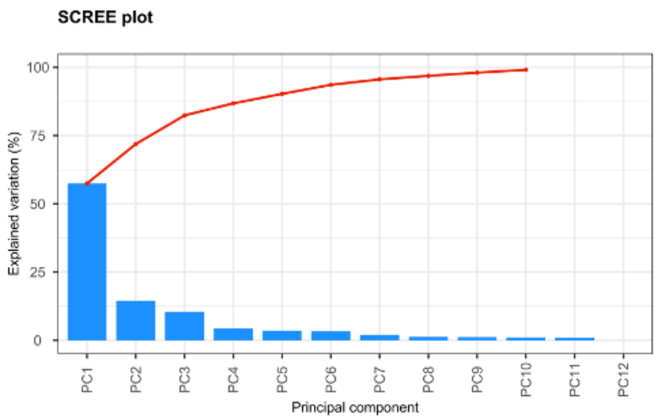

Figure S1. Validation of chicken RNAseq data. (a) Colorimetric Principal-Component Analysis (PCA) plot for samples labeled according to tissue (red: dorsal; blue: fissure) and condition (circle: HH St30; triangles: HH St34). PC1 is plotted against PC2 (n=3 for each tissue region and condition; total = 12 samples). (b) SCREE plot showing PC1 markedly reports difference between HH.St 30 versus HH.St34 samples, whereas PC2 separates fissure and dorsal tissue samples.

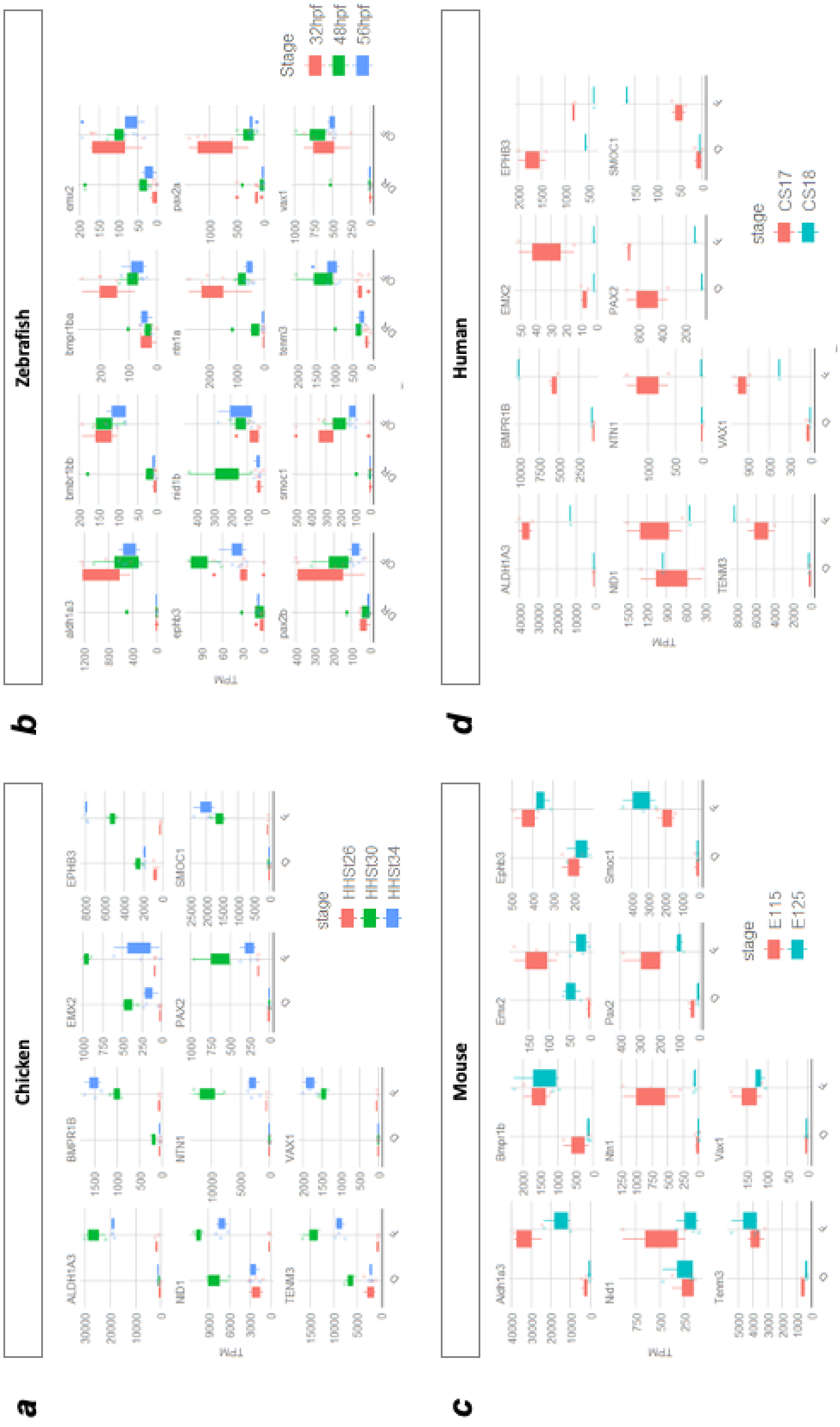

Figure S2. Expression values for all species and stages in the OFM and dorsal retina. TPM, transcripts per million; D, dorsal; F, fissure.

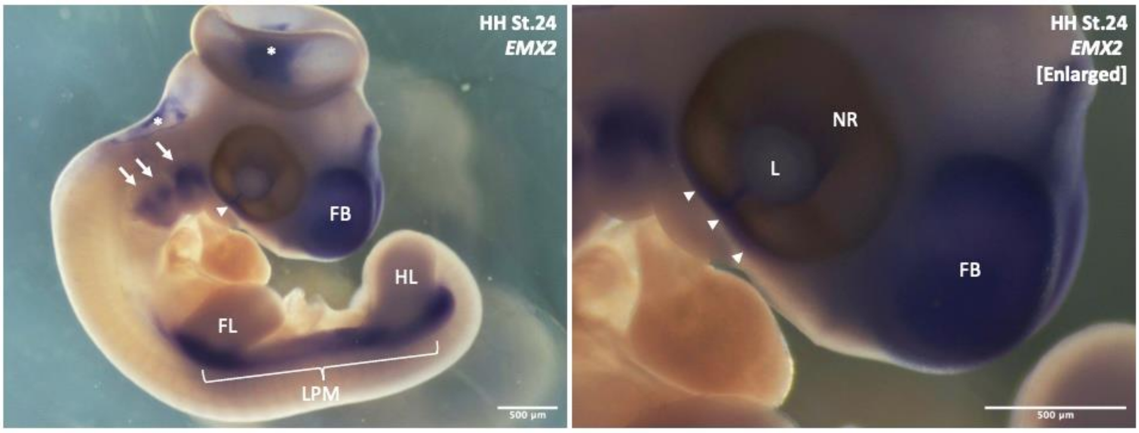

Figure S3. Colorimetric whole mount *in situ* hybridization for *EMX2* expression in HH St24 chicken embryo. Arrowheads indicate periocular mesenchyme region. Arrows indicate pharyngeal arch regions. FB, forebrain; HL, hindlimb; FL, forelimb, LPM, lateral plate mesoderm region; L, lens; NR, neural retina. Asterisks indicate artifactual staining from substrate trapping. Scale bar = 500 µm.

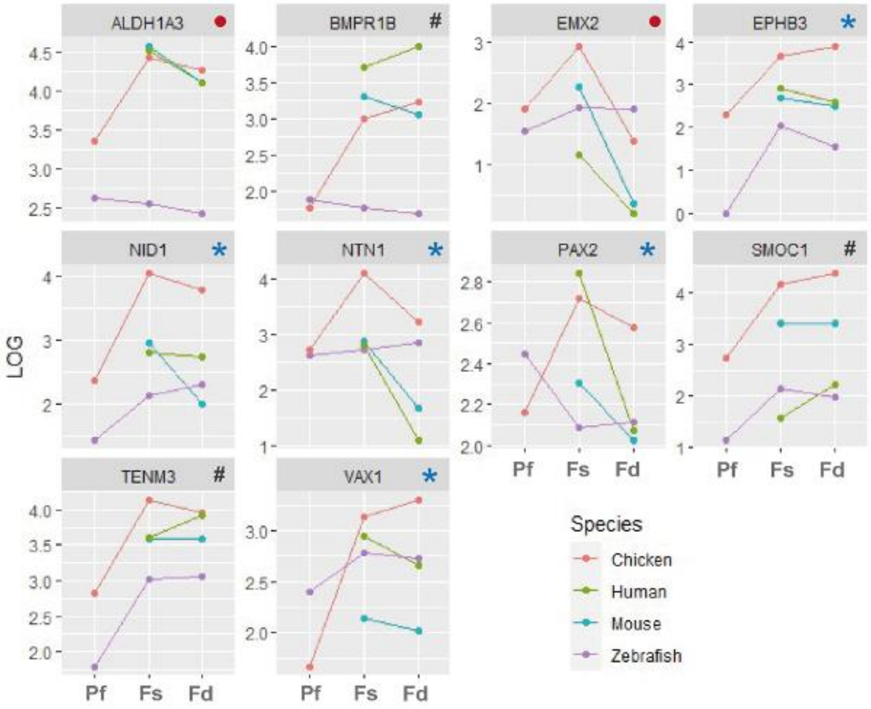

Figure S4. Expression trajectories in the OFM based on RNAseq expression data for humans, mouse, chicken, and zebrafish. Red dots indicate genes with reduced fissure expression post-fusion. Hatching indicates genes with increased or constant levels of expression post-fusion, and blue asterisks are genes with decreased fissure expression in the majority (3 of 4) of species.

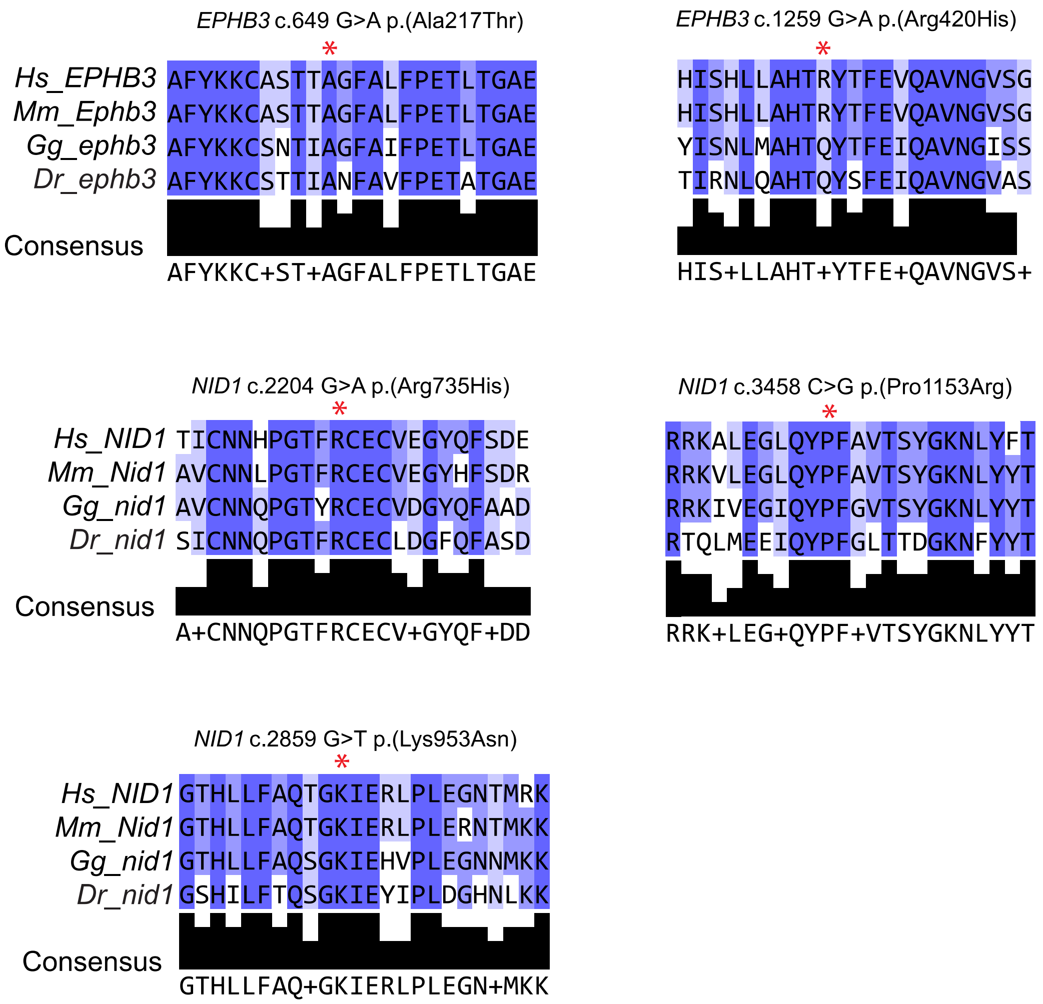

Figure S5. Multi-species amino acid alignments for each variant from Table 3. Variants are indicated by red asterisk. .
